# Discordant fetal anomalies in monochorionic multiple pregnancies

**DOI:** 10.1515/medgen-2026-2011

**Published:** 2026-04-16

**Authors:** Heidrun Schuligoi, Herbert Juch, Philipp Klaritsch

**Affiliations:** LKH Oststeiermark Department of Anesthesia and Intensive Care Medicine Ottokar-Kernstock-Str. 18 8330 Feldbach Austria; Medical University of Graz Department of Cell Biology, Histology and Embryology, and D&R Institute of Human Genetics Neue Stiftingtalstr. 6 8010 Graz Austria; Medical University of Graz Department of Obstetrics and Gynaecology, Research Unit for Foetal Medicine Auenbruggerplatz 14 8036 Graz Austria

## Abstract

Monozygotic (MZ) twins sharing a placenta are known as monochorionic (MC) and account for approximately two-thirds of MZ twin pregnancies. MC twins discordant for a congenital anomaly are of particular interest as they challenge the common idea of “identical” monozygotic twins.

We have performed a retrospective study including 51 monochorionic multiple pregnancies with discordant anomalies that received care at the University Hospital of Graz between 2013 and 2023, aiming to assess the accordance of the observed cases with the existing explanatory models in the literature. This study primarily identified structural discordance, while genetic insights were limited due to the rare decision of parents to undergo further genetic testing. The most frequent anomalies were hydrocephalus, neural tube defects, body-stalk anomaly and congenital heart defects. Acknowledging the limitations related to the sample size and incomplete cohort data, this study supports a growing awareness of the complexity underlying MC twin development, emphasizing the need for more longitudinal genetically and epigenetically focused studies to uncover the subtle and cumulative effects of early contributing factors that ultimately also determine phenotypic expression.

## Introduction

**Figure 1:**
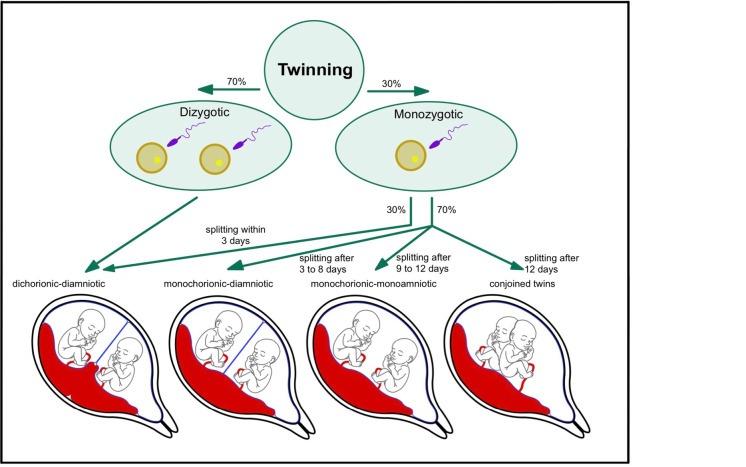
This figure depicts the relationship between the timing of embryonic splitting and subsequent chorionicity and amnionicity. The later this occurs, the more structures are shared between the twins.

Monozygotic (MZ) twins sharing a placenta are known as monochorionic (MC) and account for approximately two-thirds of MZ twin pregnancies [1]*.* The process of twinning, including the formation of the different possibilities of chorionicity and amnionicity configuration can be taken from figure 1. A shared placenta with its vascular anastomoses is often accompanied by complications such as the Twin-To-Twin Transfusion Syndrome (TTTS), thus increasing the risk of foetal and perinatal morbidity and mortality [2, 3]. However, the extraordinary unity of MC twins offers an opportunity to explore the influence of both genetic and environmental factors on human development [4].

Monozygotic twins are considered “genetically identical”; however, this assumption may be challenged when prenatal examinations reveal MC twins to be both structurally and/or genetically discordant. There are several reports in the literature on discordant structural and genetic anomalies in MZ twins as well as differences in phenotypes in monogenic disorders. This reveals the complexity underlying this phenomenon. For instance, in some cases of body stalk anomaly, colour-blindness, Beckwith-Wiedemann syndrome, Duchenne muscular dystrophy, haemophilia A and fragile X syndrome, only one of the MZ twins developed the disorder [5, 6, 7, 8, 9, 10].

Observed discordant structural malformations in MC twins often include congenital heart defects (CHD), with a reported incidence of 59 per 1000 live births [11]. While CHD-specific mutations in discordant twins are rarely identified, differentially methylated regions in the promoters of genes related to cardiac development were identified in some of them [11]. However, since the relationship between gene expression and epigenetic modifications may be interdependent, it remains unclear whether these differentially methylated regions are the cause or the consequence of developmental processes. While maternal co-morbidities such as diabetes, hypertensive disorders, or pre-pregnancy BMI appear to have no impact, given that they affect both twins equally, placenta related pathophysiology seems to play a more substantial role. One study reported placenta-related pathophysiology in 41 % of all discordant CHD cases. In TTTS cases, the frequency of CHD increases to 9.3 % [11]. However, these reports did not differentiate between CHD and heart problems caused by abnormal circulation in TTTS. Consequently, relative hypoperfusion of one twin has been suggested as a possible cause of discordance in CHD [11].

In the present study, we examined cases of MC twins with discordant structural and genetic anomalies in multiple pregnancies treated at the Department of Obstetrics and Gynaecology at the Medical University of Graz between 2013 and 2023. These were put into relation with previously reported cases. Furthermore, some of the most intriguing hypotheses regarding the pathogenesis are highlighted.

## Hypotheses on the genesis of discordance

Some experts suggest that genetic or structural discordance does not arise after embryonic splitting but may serve as the primary trigger for twinning [12]. In this context, the unique environment of a single shared placenta must be acknowledged. The amount of placenta allocated to each twin may differ, as may the number of cells allocated, which may be an underlying cause of discordance [13]. Additional hypotheses extend this concept, including disproportional blastomere distribution, X-inactivation, chromosomal mosaicism, and intermediate twins.

### Unequal blastomere distribution

A disproportion in the distribution of blastomeres during the twinning process would imply that they begin to diverge in their development from the moment of twinning, thus laying the foundation for further differences [12]. A pair of discordant female twins inspired this hypothesis. One twin exhibited the expected random X-inactivation, while the other demonstrated a skewed X-inactivation [14].

Unequal blastomere distribution could also have vascular implications. A twin with fewer initial cells might receive a smaller portion of the shared placenta, leading to growth impairments. As a result, the intrauterine environment of MC twins may be even less similar than that of dizygotic (DZ) twins.

### X-inactivation and imprinting

Cases of skewed X-inactivation in MC twins have been described, including haemophilia A or fragile X syndrome [9, 10, 15]. The healthy twin showed either random X-inactivation or an inactivation of the mutated X chromosome, while the affected twin displayed non-random inactivation of the X chromosome carrying the wild type gene [12]. Two possible mechanisms are proposed to explain these findings. First, within the inner cell mass, two clones of cells with dissimilar X-inactivation patterns may segregate and demonstrate mutual aversion from the clones with inverse inactivation pattern. The X-inactivation would proceed and may subsequently trigger the splitting, ultimately resulting in a reciprocal skewed X-inactivation [12]. However, this remains a highly speculative scenario, implying the existence of individuals with exclusively maternal or paternal X-inactivation, which has never been documented so far, in MC twins. Another possibility entails a random X-inactivation but draws back to the unbalanced allocation of blastomeres. It is then likely that the twin with the smaller portion of blastomeres would conclude with a higher likelihood of skewed X-inactivation, whereas the co-twin would be more likely to experience balanced inactivation. Machin suggested that if skewed X-inactivation was indeed a common occurrence in MC twins, it might even represent a crucial factor in the twinning process, possibly even playing a role in its initiation. This could account for the observed higher number of females among MZ twins. It was even suggested that singleton females with secondary skewed X-inactivation and resulting X-linked diseases may represent surviving twins of MZ pairs [12].

### Chromosomal mosaicism

As a post–zygotic genetic event, chromosomal mosaicism has also been considered to account for genetic and structural discordance in MZ twins. In cases with twins with discordant expressions of aneuploidy-phenotypes, such as Turner Syndrome, trisomies 21, 18 and 13, chromosomal mosaicism has been identified [[12][16]]. In the literature there are also reports on MZ twins with discordant karyotypes of 45,X and 47,XXY, as well as a female pair with a 45,X and 47,XXX cell line, in neither a 46,XY or 46,XX cell line was detected [17, 18]. Non-disjunction may be the underlying cause.

Additional instances of non-disjunction were observed in MZ twin pairs discordant for trisomy 21, who presented with 46,XY/47,XY+21 mosaicism in blood but were non-mosaic in fibroblasts [19]. A different case demonstrated a MZ pair with a discordant phenotype of the fragile X syndrome with discordant trinucleotide expansion. The unaffected twin was a mosaic for the premutation, while the affected twin carried the full mutation [10]. Mosaicism for structural chromosome anomalies was also described. Various examples, such as a MZ pair discordant for a 46XY,del(7)(q32->qter) chromosome constitution with mosaicism in lymphocytes are reported in the literature [20].

## Materials and methods

### Research question

We conducted a retrospective analysis of MC multiple pregnancies with discordant anomalies from 2013 to 2023 to assess the degree of concordance between observed cases and the existing explanatory models in the literature. This was a descriptive, retrospective, and monocentric study conducted at the Department of Obstetrics and Gynaecology at the Medical University of Graz. All data were pseudonymized to ensure patient confidentiality.

### Data collection

All data were obtained from the local registry “MonoReg”, maintaining records of patients with multiple pregnancies at the University Hospital in Graz, Austria. The registry had received prior approval from the ethics committee (EK 29-105 ex 16/17). Patient information (medical reports, clinical notes and diagnostic findings) was retrieved from medical records in the hospital information system, including “openMEDOCS” and “Pia ViewPoint”. Data was systematically extracted to complete the following categories:

Patient demographics: date of birth
Pregnancy characteristics: chorionicity/amnionicity, foetal anomaly, genetic findings (if available)
Diagnosis and intervention details: gestational age at diagnosis, type of intervention, gestational age and date at intervention
Pregnancy outcome: gestational age at birth or miscarriage, date of birth/miscarriage, location of referral
Maternal factors: artificial reproductive technologies (ART), pre-existing medical conditions

**Figure 2:**
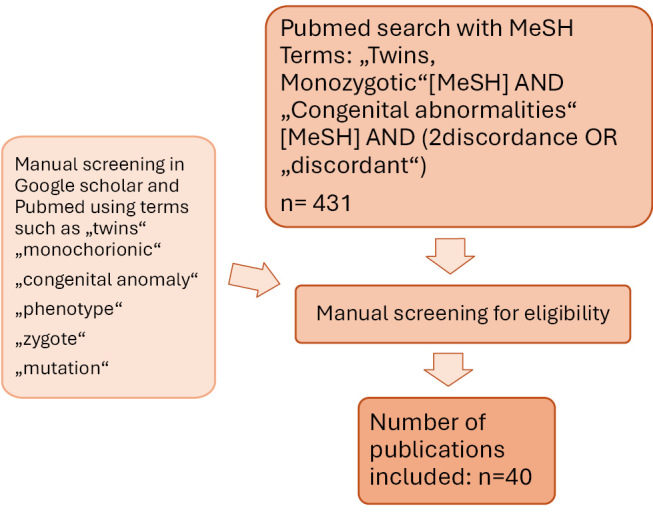
A schematic overview of the types of materials utilised and analysed during the literature review

However, not all categories could be completed for every patient. Information on genetic findings was limited, as not all patients underwent genetic testing, and in some cases, results were restricted due to confidentiality regulations. Moreover, 44 patients were only referred to the hospital for consultation and potential treatment, while continuing their prenatal care and delivery at their local hospital. Inclusion in the the “MonoReg” registry, required care at the Department of Obstetrics and Gynaecology in Graz for a multiple pregnancy with either genetic discordances, such as differing karyotypes, or structural discordance, defined as the presence of a congenital anomaly in one foetus but not the co-twin, as confirmed by prenatal imaging or invasive genetic testing. All women were required to be at least 18 years old. Triplet pregnancies were included, provided that at least two of the three foetuses shared a MC placenta. A comprehensive literature search was conducted using PubMed, the applied search strategy is illustrated in figure 2

**Table 1:**
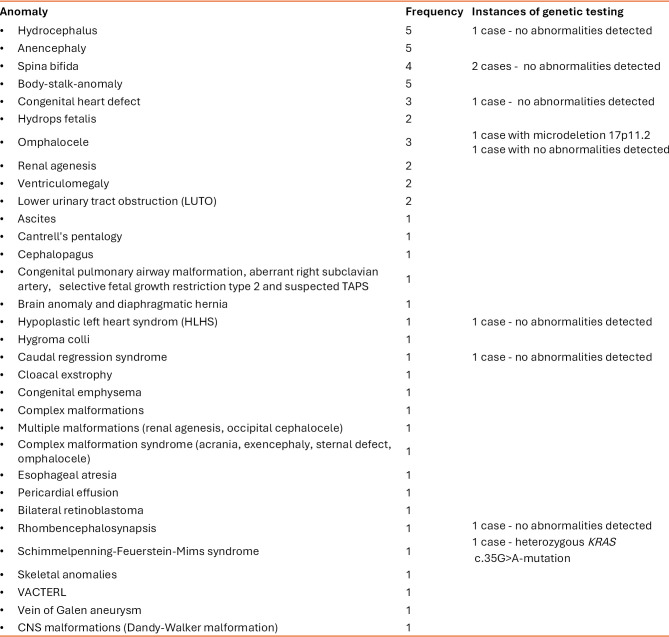
Overview of the anomalies in a cohort of 51 discordant MC twins. The cases were collected in Graz between 2013 – 2023. Individuals with more than one anomaly are represented multiple times. Genetic testing was performed in ten cases.

## Results

We included 51 patients. Maternal age ranged from 20 to 43 years. An overview of the discordant anomalies is provided in table 1.

Out of the 51 MC twins with discordant anomalies, 52.9 % (n=27) underwent selective feticide (in accordance with the Austrian law) by cord occlusion (CO) at parental request. Radiofrequency ablation and laser coagulation were performed in four cases each (7.8 %), while conservative management was chosen in 29.4 % (n=15). Potassium chloride injection was used in only one single case (n=1), following prior laser ablation of communicating vessels.

In this cohort, 88.2 % of pregnancies were conceived spontaneously, while 11.8 % resulted from (ART). In most cases (86.3 %), no maternal comorbidities were reported. Among the remaining patients, obesity (BMI ≥ 30) was the most frequently documented condition (5.9 %), followed by single cases of gestational hypertension, Turner syndrome (pregnancy conceived following egg donation), factor VII deficiency and hypothyroidism.

In the predominant proportion of cases (90 %), neither TTTS nor Twin Reversed Arterial Perfusion (TRAP) sequence was observed. TTTS was diagnosed in 7.8 %, while Twin Anemia Polycythemia Sequence (TAPS) sequence occurred in a single case. These findings indicate that severe placental vascular complications were relatively rare in this cohort.

Genetic testing was offered to the families, with ten choosing to proceed with genetic analysis of the affected twin. Among these, eight cases revealed no detectable genetic abnormalities. In one case, a postnatally detected heterozygous KRAS c.35G>A mutation, representing a postzygotic event causing somatic mosaicism, resulted in the discordant clinical phenotype of Schimmelpenning-Feuerstein-Mims syndrome. Another case with discordant omphalocele revealed a microdeletion on chromosome 17p11.2 (Smith-Magenis-Syndrome), which was absent in the co-twin.

## Discussion

The literature describes a broad spectrum of anomalies for which MC twins have been found to be discordant. Explanations such as skewed X-chromosome inactivation have been proposed as potential mechanism, especially considering that the literature reports a higher incidence of female monozygotic twins. However, many hypotheses predominantly focus on MZ twins, neglecting and not specifically addressing MC twins, thereby overlooking the distinct placental and vascular environment of MC pregnancies. While some publications have considered the unique vascular situation, this has largely been examined in the context of CHDs in discordant MC twins, with TTTS often proposed as a major contributor to discordance. However, in our cohort, TTTS or TAPS was diagnosed in only five cases, none of which was associated with CHD. This may challenge the hypothesis that such vascular complications are a major driver of discordant anomalies. Nevertheless, given the limited sample size of this cohort, these results must be interpreted with caution.

There is an ongoing discussion regarding whether maternal age influences the rate of MC twinning. One study indicating that a higher maternal age is not a characteristic feature of MC twinning examined a cohort of MC twins with discordant anomalies in which the mean maternal age was 27.9 years, compared to 30.9 years for DC twins [21]. Both groups are, however, below the age that is considered to be advanced maternal age. Regarding our cohort, 30 years was both the median as well as the mean maternal age, representing the average maternal age at childbirth across the European Union in 2022, which was 30.9 years [22]. Given this, our findings do not show an overrepresentation of either younger or older maternal age among MC twin pregnancies, thereby failing to support either side of the conflicting views reported. This suggests that maternal age alone may not be a reliable predictor of chorionicity.

CHD was among the more frequent anomalies in our cohort (5.8 %), although this finding must be interpreted with caution, as CHD represents the most commonly reported congenital anomaly in Europe. Thus, its occurrence in our cohort may simply reflect its high baseline prevalence [23]. Nonetheless, the previously discussed theories have proposed that the vascular and hemodynamic environment of MC twins may contribute to an elevated risk of CHD. Hence, while a direct association cannot be established based on our data, the interaction between general population risk factors and MC-specific mechanisms calls for further investigation.

Interestingly, the central nervous system (CNS) is affected the most frequently in MC twins [21], especially by neural tube defects (NTD) [24]. Consistent with this observation, anencephaly and spina bifida were among the most prevalent anomalies in our cohort. Notably, when compared to the EUROCAT registry, NTDs were ranked only 16th in frequency, while hydrocephalus, the most common anomaly in our cohort, was ranked 15th. This discrepancy suggests that CNS anomalies may occur disproportionately more often in MC twin pregnancies compared to the general population. In a cohort of 108 discordant MC twins 49,1 % showed CNS abnormalities [25]. Rustiko et al. describe CNS anomalies in 54 of 155 discordant MC pregnancies (34.8 %) [26, 27]. In comparison, globally the rate is estimated to be approximately two per 1000 births [28].

Even though ART is known to elevate MZ twinning, its effect on MC twinning is less certain. In our cohort, 12 % of pregnancies were conceived using ART, indicating that ART does not seem to have played a substantial role.

The literature mainly describes that so far, ART is particularly associated with a higher rate of DZ twinning, largely due to the implantation of multiple embryos during IVF cycles and the stimulation of ovaries, leading to the release of multiple oocytes [29]. The practice of extended embryo culture and blastocyst-stage transfers has been proposed as a factor contributing to a higher risk of MZ twinning [30].

In discussing discordant anomalies in MC twins, it is necessary to differentiate between monogenic and multifactorial conditions. Thereby also acknowledging that only a fraction of human diseases is predominantly monogenic. Assuming that genetic differences are not the primary trigger for splitting, and that both MZ twins start with the same genetic constitution, from the moment of separation onward, each twin embarks on its own unique developmental and environmental journey. This may include epigenetic changes, unequal placental sharing, and vascular anastomoses, all of which can lead to asymmetric intrauterine exposures. Therefore, despite their shared genetic origin, MC twins do not necessarily represent an identical combination of genetic and environmental factors, contributing to phenotypic discordance, including a discordant manifestation of congenital anomalies [31].

Multifactorial diseases are thought to operate under a liability threshold model [31]. This model postulates that an accumulation of predisposing factors, genetic or environmental, must be surpassed for a condition to manifest. While the foundational genetic loci involved may still adhere to Mendelian inheritance, the multifactorial model introduces additional principles. For instance, it proposes that multiple genetic loci interact together, with each locus contributing to or taking from the likelihood of the trait’s manifestation. Additionally, the model acknowledges the significant influence of environmental factors in shaping the phenotype. Consequently, the interplay of these numerous genetic loci and environmental factors creates a spectrum of potential phenotypes. This variability helps to explain why even genetically near-identical MC twins, experiencing differing intrauterine environments and thus potentially accumulating a different combination and quantity of these contributing factors, can present with markedly different clinical phenotypes, with one twin potentially crossing the liability threshold for a specific anomaly while the other does not.

This concept is further emphasised by the unique intrauterine environments that MC twins are exposed to, particularly those who develop vascular anastomoses within their shared placenta, which is thought to be the case in the vast majority. These vascular connections can lead to an unbalanced blood flow, further supporting the likelihood that environmental factors do not uniformly accumulate in both twins and, consequently, may not result in the same phenotypic outcome.

Considering the liability threshold of multifactorial disorders, along with the most frequent cases observed in our cohort, it becomes apparent that these conditions are known as multifactorial disorders. For example, congenital hydrocephalus serves as an example of a multifactorial disorder where genetic and environmental factors are known to be involved in determining its manifestation [32]. It remains questionable whether the observation of discordant congenital hydrocephalus in MC twins, especially in the absence of typical complications like TTTS, TAPS or selective foetal growth restriction (sFGR), could therefore be rather explained by the aleatory surpassing of a liability threshold in one of the twins than by an obvious pathological factor caused by the MC state itself.

Cases of NTDs were observed in the cohort in Graz. It is unclear what exactly was causing it. Although as a typical malformation with a multifactorial aetiology, it is thought that a combination of predisposing genotype and environmental factors influences neural tube closure [33]. The higher incidence of NTDs in twins is well known, with the risk being particularly elevated in MC twins [24, 34]. For example, one study reported that among all investigated twin pregnancies affected by NTDs, 68 % involved MZ twins [34]. This proportion was considered excessive given that MZ twins are rarer than DZ twins.

CHD is also widely recognized as a multifactorial disorder and represents the most common type of birth defect, affecting almost one percent of live births. Around 100 genes have been associated with various forms of CHD [35]. Nevertheless, large-scale whole-genome sequencing studies have identified a clear genetic cause in only about 30 % of cases, highlighting that a purely monogenic explanation is insufficient in more than two-thirds of the cases, suggesting that in addition to possible polygenic genetic risk factors, environmental factors play a substantial role in the aetiology [36]. In the context of twin pregnancies, additional complexities arise. A study observed that concordant CHDs appeared more frequently in same-sex twin pairs (6.1 %) than in opposite-sex twins (3.3 %) [37]. While zygosity and chorionicity were not explicitly reported, it was presumed that all MZ twins are in the same-sex cohort, raising questions about genetic and shared environmental influences. However, the lack of precise classification limits the strength of such conclusions, particularly in relation to MC twins. An earlier study on CHD in twins reported on a cohort of 26 twins. Each pair was discordant for CHD and 13 of them turned out to be MZ twins [38]. Such observations emphasize the importance of considering each twin as an individual case, rather than assuming a shared causative mechanism. Other reports have echoed these findings, suggesting that while genetic background is important, individual intrauterine conditions may have an even greater impact on CHD manifestation in twins [11, 39].

### Limitations

The interpretation of our findings must be considered within the context of the following limitations. As MC twins discordant for genetic or structural anomalies represent a particularly rare and highly specialised subset, sample sizes in studies found in the literature, as well as our own, are limited. Due to these small case numbers, studies often lack sufficient statistical power to draw definite conclusions.

Concerning the presented data, data on detailed pregnancy outcomes were frequently unavailable, a limitation that has been repeatedly identified in studies of MZ twins [40]. Many patients decline genetic investigations for personal or cultural reasons, and even when testing is performed, access to detailed results is often highly restricted by confidentiality policies. However, genetic analyses are crucial for identifying potential underlying genetic or chromosomal abnormalities that may contribute to the observed anomalies. In the absence of comprehensive genetic data, the ability to interpret the aetiology, distinguish between genetic and environmental contributions, and systematically assess genetic discordance in MC twins remains limited.
